# Identification of Important Modules and Hub Gene in Chronic Kidney Disease Based on WGCNA

**DOI:** 10.1155/2022/4615292

**Published:** 2022-05-04

**Authors:** Jia Wang, Yuan Yin, Qun Lu, Yan-rong Zhao, Yu-jie Hu, Yun-Zhao Hu, Zheng-Yin Wang

**Affiliations:** Clinical Laboratory, Shanghai Traditional Chinese Medicine-Integrated Hospital, Shanghai, China

## Abstract

Chronic kidney disease (CKD) is an ongoing deterioration of renal function that often progresses to end-stage renal disease. In this study, we aimed to screen and identify potential key genes for CKD using the weighted gene coexpression network (WGCNA) analysis tool. Gene expression data related to CKD were screened from GEO database, and expression datasets of GSE66494 and GSE62792 were obtained. After discrete analysis of samples, WGCNA analysis was performed to construct gene coexpression module, and the correlation between the module and disease was calculated. The modules with a significant correlation with the disease were selected for Gene Ontology (GO) and the Kyoto Encyclopedia of Genes and Genomes (KEGG) enrichment analysis. Then, the interaction network of related molecules was constructed, and the high score subnetwork was selected, and the candidate key molecules were identified. A total of 882 DEGs were identified in the screening datasets. A subnetwork containing 6 nodes was found with a high score of 12.08, including CEBPZ, IFI16, LYAR, BRIX1, BMS1, and DDX18. DEGs could significantly differentiate CKD and healthy individuals in principal component analysis. In addition, the MEturquiose, MEred, and MEblue in group were significantly correlated with disease in WGCNA. These 6 hub genes were found to significantly discriminate between CKD and healthy controls in the validation dataset, suggesting that they could use these molecules as candidate markers to distinguish CKD from healthy people. Overall, our study indicated that 6 hub genes may play key roles in the occurrence and development of CKD.

## 1. Introduction

Chronic kidney disease (CKD) is a worldwide public health problem with increasing incidence, poor prognosis, and high treatment cost. As a global health problem, CKD affects 10-16% of adults in Asia, Europe, and the United States and can progress to kidney failure [1]. The development of disease-related modules and genes is becoming increasingly popular. These methods are extremely useful in aiding the clinical search for diagnostic and therapeutic indicators. CKD is a complex disease related to genetic and environmental risk elements [2–4]. In response to the growing need to identify patients with CKD at an early stage and improve risk stratification for progression to end-stage renal disease, numerous studies have been conducted in large numbers of patients to investigate new and existing kidney disease biomarkers.

A number of different methodologies have been used, ranging from candidate single-gene studies to genome-wide multiomics analyses, to identify potential drug candidates. It is hoped that new methodologies will be developed to discover new CKD biomarkers, which will help us better understand the biology of kidney disease by using genetic, epigenetic, and transcriptome investigations [5]. CKD is an important issue given the increasing number of such patients worldwide. CKD is characterized by glomerular filtration rate (GFR) of less than 60 mL/min/1.73 m^2^ and signs of renal injury lasting at least 3 months. Reduced estimated GFR (eGFR) and severity of proteinuria independently predicted end-stage renal disease and mortality in patients with CKD [6, 7]. There is an urgent need to identify new biomarkers in patients with CKD to better detect people at high risk of rapid decline in kidney function, so that effective therapies can be used to curb disease progression [8, 9].

Weighted gene coexpression network analysis (WGCNA), as a method to screen disease-related modules, is the most common and useful method for discovering the link between genes and clinical characteristics [10]. Complex disorders including glioblastoma multiforme, cardiovascular disease in patients with diabetes, and Sjogren's syndrome have been studied using WGCNA in prior studies [11–13]. WGCNA analysis provides an alternative approach for exploring genetic biomarkers that predict prognosis for CKD. The expressing data can be used to construct significantly correlated genes and their coexpression modules. In addition, these modules can further analyze modular characteristic genes (ME) and intramodular hub genes [14, 15]. Therefore, WGCNA may be a valuable tool for a comprehensive understanding of CKD-related genomic changes [10]. However, there was still little researches on CKD. In this study, WGCNA was performed to identify and screen related genes, so as to further explore the possible mechanisms of the critical genes and provide candidate markers for the diagnosis of CKD.

## 2. Materials and Methods

### 2.1. Microarray Data Source

The information was from the GEO datasets for patients with CKD extract (https://www.ncbi.nlm.nih.gov/gds/). The keywords “Chronic kidney disease” and “Homo sapiens” were applied as queries to search ckD-related datasets from GEO datasets. The GEO dataset met the following criteria: (1) the dataset contains CKD specimens and normal specimens; (2) each sample was assigned a group label; (iii) platform type is limited to “microarray”; (4) each probe has an available gene symbol or GeneBank ID; (5) the number of samples in the dataset was greater than 10. Finally, two datasets including GSE66494 and GSE62792 were selected as analysis datasets, including 65 CKD patients and 14 healthy controls.

### 2.2. Identification of Differentially Expressed Genes (DEGs)

In order to find differences in gene expression between CKD samples and healthy controls, GEO2R was used [16]. The log-fold changes in expressions and adjusted *P* values (adj.*P*) were calculated. The adj.*P* was corrected for false-positive results using the Benjamini-Hochberg method and default settings. An adj.*P* 0.05 and ∣logFC | >2 cut-off was used to identify the DEGs. A Venn diagram web tool was used to identify genes that overlapped. In order to see the DEGs volcano plot visually, a hierarchical cluster analysis was performed.

### 2.3. Clustering and Enrichment Analysis

Clustering and enrichment analyses were carried out using GO (Gene Ontology) analysis. Pathway analysis is described in the Kyoto Encyclopedia of Genes and Genomes (KEGG). Enrichment analysis uses DAVID (https://david. http://ncifcrf.gov/tools. jsp), including biological process, molecular function, cells, and KEGG analysis. In addition, only FDR of GO or KEGG terms less than 0.05 was considered significant. We carried out visualization of the top 10 GO terms and the top 10 KEGG pathways.

### 2.4. Weighted Gene Coexpression Network Analysis

We compile and organize data files containing gene expression and phenotypes in standard formats. Firstly, to validate the accuracy of the study, we performed sample cluster analysis to verify the association of all data in the training queue. As a mean of ensuring that gene interaction followed a scale-free distribution, a study known as soft threshold selection analysis was employed. Besides, dynamic tree cutting algorithm was applied to identify modules via hierarchical clustering. Then, the protein expression abundance was clustered in R software (https://http://www.r-project.org/) to construct a weighted gene network. Subsequently, the correlation and correlation coefficient between the expressing spectrum and groups were calculated. We further identify important modules associated with traits. The dissimilarity degrees of MES in the module tree were calculated, and some modules (dissimilarity degree of MES < 0.25) were combined to obtain the final network.

### 2.5. Construction of an Interactive Network

The molecular network was based on the interaction of text mining, experiment, database, coexpression, neighborhood, gene fusion, cooccurrence, and so on. Molecular networks and subnetworks were optimized for social networks constructed by STRING (https://http://string-db.org/cgi/input.pl) and Cytoscape software (version 3.7.2). Cytoscape software was used to depict the results from STRING as PPI networks. The PPI network was cleansed of nodes that were not connected to any other nodes. Degree centrality was used to determine the PPI network's hub genes. The subnetwork was analyzed and scored using Cytoscape's MCODE plug-in.

### 2.6. Statistical Analysis

Statistical analysis was performed by SPSS software (SPSS Inc., Chicago, IL, USA) and R.4.1.1 (R Core Team, Massachusetts, USA). Data visualization was performed using PRISM. The differential expression threshold was set to 1.5. *T*-test was used to calculate the difference, and *P* < 0.05 was considered statistically significant.

## 3. Results

### 3.1. Differential Expression Analysis in CKD Patients

**Figure 1:**
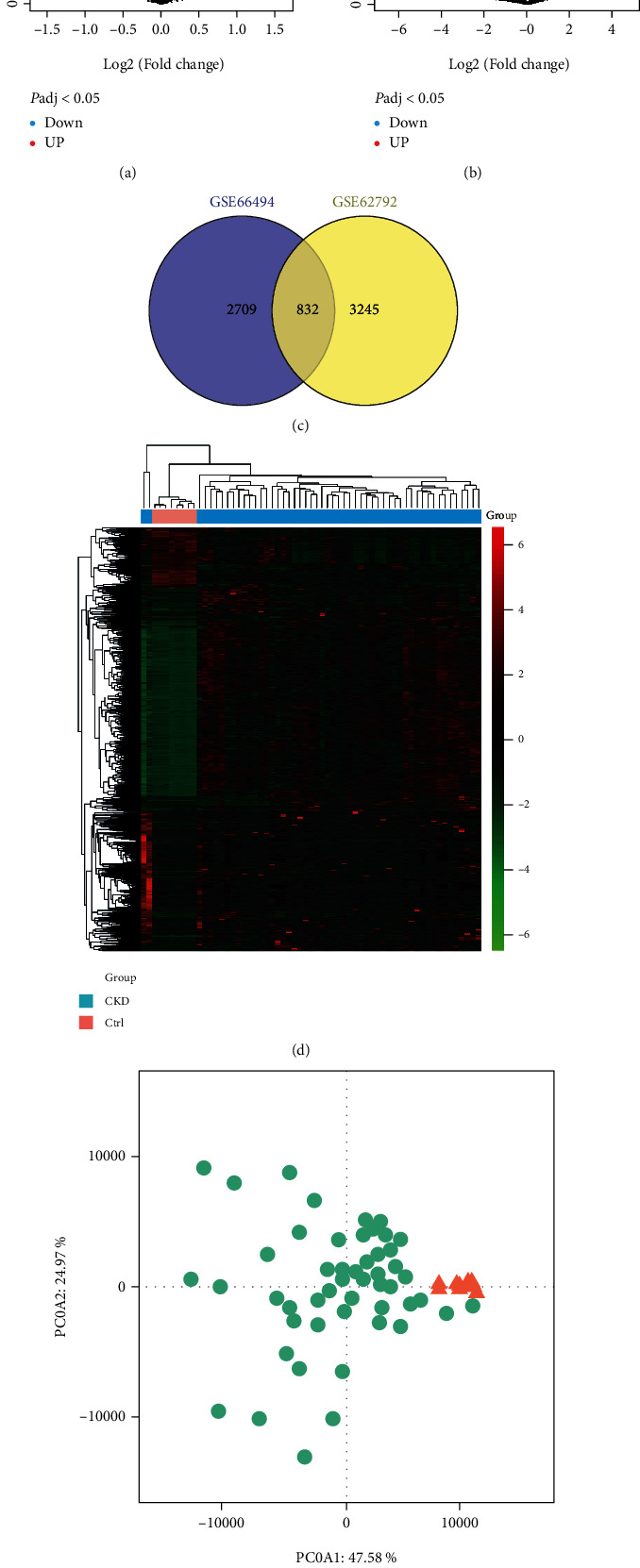
Differential analysis of gene expression profiles. (a) The volcano map of GSE62792. (b) The volcano map of GSE66494. (c) The Venn diagram of GSE62792 and GSE66494. (d) The heatmap of differential expression. (e) Principal component analysis.

First, we screened gene expression profiling datasets from CKD patients and healthy individuals in the GEO database. A total of renal biopsy specimens of CKD patients were selected for microarray analysis data, and datasets GSE66494 and GSE62792 were selected as analysis datasets, including data of 65 CKD patients and 14 healthy controls. Data were obtained from the platform Agilent-014850 Whole Human Genome Microarray 4x44K G4112F (Probe Name version) and stored in the GPL6480 platform. We calculated DEGs between CKD patients and healthy individuals (Figures 1(a) and 1(b)). We found that a total of 882 DEGs were screened out in the above two datasets (Figure 1(c)). We found that a total of 882 DEGs were screened in the above two datasets, and their gene expression profiles were listed in the heatmap (Figure 1(d)). Additionally, DEGs were observed to significantly distinguish CKD patients from healthy controls in principal component analysis (Figure 1(e)).

### 3.2. Clustering and Enrichment Analysis

**Figure 2:**
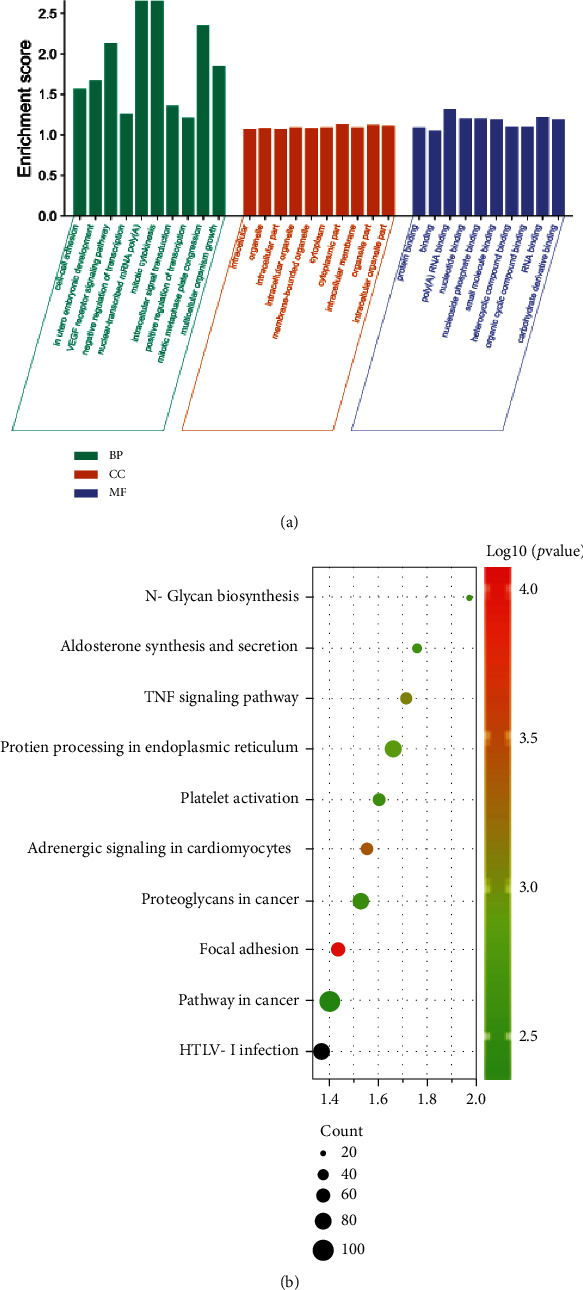
GO analysis and KEGG enrichment analysis. (a) GO enrichment analysis of differentially expressed genes, including biological processes, cellular components, and molecular functions; (b) KEGG pathway enrichment skin run map of related molecules.

In order to further learn the function of CKD-related genes, we conducted GO function and KEGG pathway enrichment analysis of related genes. GO enrichment results showed that, for BP, these related molecules mainly play physiological functions including cell-cell adhesion, in utero embryonic development, VEGF receptor signaling pathway, negative regulation of transcription, nuclear-transcribed mRNA poly (A), mitotic cytokinesis, intracellular signal transduction, positive regulation of transcription, mitotic metaphase plate congression, and multicellular organism growth (Figure 2(a)). The results showed that for CC, these related molecules were mainly located in the intracellular, organelle, intracellular part, intracellular organelle, membrane-bounded organelle, cytoplasm, cytoplasmic part, intracellular membrane, organelle part, intracellular organelle part (Figure 2(a)). The results showed that, for MF, the main physiological functions of these related molecules included protein binding, binding, poly (A) RNA binding, nucleotide binding, nucleoside phosphate binding, small molecule binding, heterocyclic compound binding, organic cyclic compound binding, RNA binding, and carbohydrate derivative binding (Figure 2(a)). In order to understand the enrichment of the pathway, KEGG analysis was used to analyze the pathway, and it was found that these molecules were mainly involved in 14 pathways including protein processing in endoplasmic reticulum, pathways in cancer, proteoglycans in cancer, TNF signaling pathway, platelet activation, aldosterone synthesis and secretion, adrenergic signaling in cardiomyocytes, focal adhesion, N-glycan biosynthesis, and HTLV-I infection (Figure 2(b)).

### 3.3. Construction of Coexpression Networks

**Figure 3:**
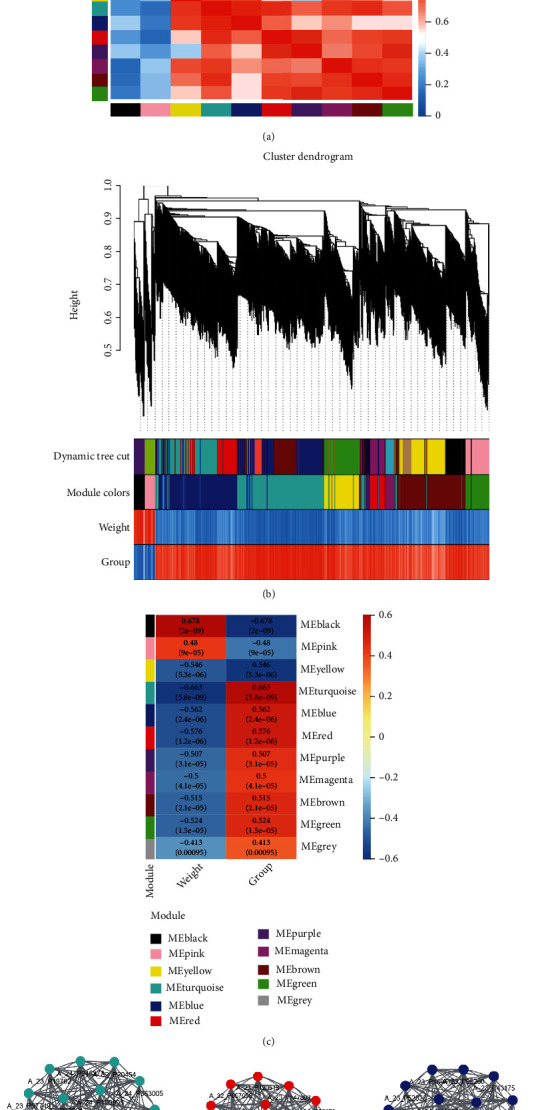
WGCNA analysis of gene expression profiles in CKD patients and healthy individuals. (a) Sample hierarchical clustering dendrogram; (b) clustering dendrogram, different color blocks represent gene modules formed by dynamic tree cutting method; (c) gene module heatmap of correlations with clinical features. (d) Interaction network analysis is performed in the turquoise module. (e) Interaction network of red modules. (f) Interaction network of blue modules.

WGCNA was used to identify disease-related modules in which genes exhibited coordinated expression patterns, which greatly improved the chances of identifying hub genes. In order to construct gene coexpression network, GSE66494 and GSE62792 data were used for cluster analysis. A total of 79 samples, including 10891 gene expression data, were used to construct hierarchical clustering trees. The analysis results showed that no obvious outlier samples were found. Thus, the analysis program retained all samples to construct the weighted coexpression network (Figure 3(a)). The dynamic mixed shearing methods were used to merge the modules with high similarity of feature genes, and 10 gene modules with different colors were finally obtained, among which the gray module was the gene without coexpression (Figure 3(b)). In addition, we also conducted hierarchical clustering of these gene modules, and these characteristic gene modules could be grouped into two categories. Disease was considered as the main clinical feature for correlation analysis of different gene modules. MEblack and MEpink were negatively correlated with the disease, while MEyellow, MEturquiose, MEblue, MEred, MEpurple, MEmagenta, MEbrown, and MEgreen were positively correlated with the disease. However, the results showed that MEturquiose, MEred, and MEblue in group were significantly correlated with disease (Figure 3(c)). Then, the MEturquiose, MEblue, and MEred were significantly positively correlated modules in the CKD and healthy groups, and it turned out that there was a complex network of connections between these molecules (Figures 3(d)–3(f)).

### 3.4. Interaction Network Analysis

**Figure 4:**
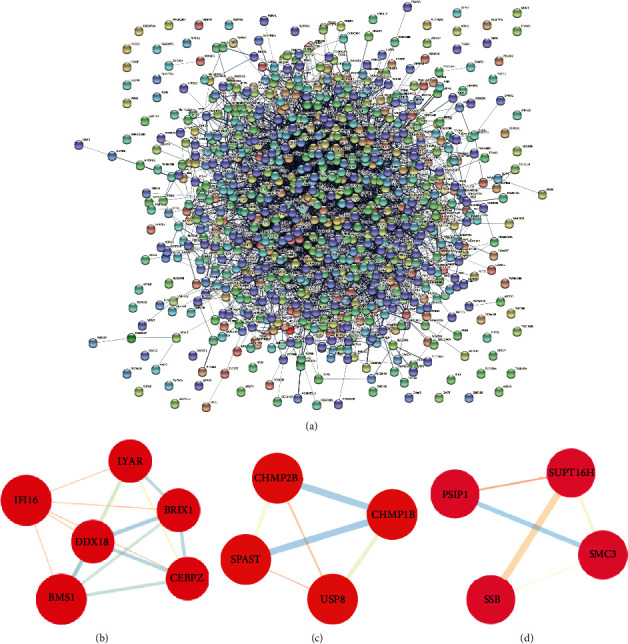
Construction of hub gene interaction networks and subsets. (a) Interaction network of related gene in 882 DEGs; (b) subnets with high scores were identified by the Cytoscape plugin MCODE. Subnets containing 6 hub genes were identified with a score of 12.08. (c) Subnets containing 4 hub genes were identified with a score of 4.01. (d) Subnets containing 4 hub genes were identified with a score of 3.33.

To understand the interaction network status of CKD-related molecules, the interaction network was constructed through STRING. It turned out that there was a complex network of connections between these molecules in DEGs (Figure 4(a)). We analyzed and extracted key subnetworks through the Cytoscape plugin MCODE. The results showed that there were 3 subnetworks with high scores, including 14 key genes in total (Figures 4(b)–4(d)). Most importantly, a subnetwork containing 6 nodes was found with a score of 12.08, including CCAAT enhancer binding protein zeta (CEBPZ), interferon gamma inducible protein 16 (IFI16), Ly1 antibody reactive (LYAR), biogenesis of ribosomes BRX1 (BRIX1), BMS1 ribosome biogenesis factor (BMS1), and DEAD-box helicase 18 (DDX18). We used these molecules as candidate markers to distinguish CKD from healthy people.

### 3.5. Validation of Candidate Markers

**Figure 5:**
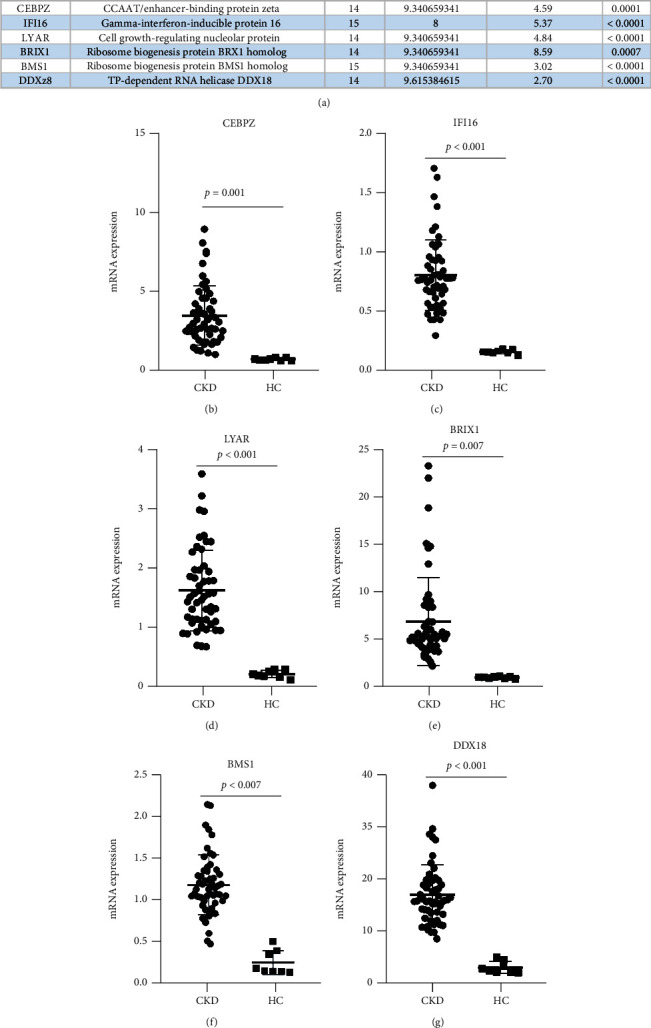
The hub genes screened out were validated in the validation dataset including GSE142153 and GSE70528. (a) A total of 6 molecules were considered to be highly associated with CKD, including CEBPZ, IFI16, LYAR, BRIX1, BMS1, and DDX18. Details of these hub genes include degree, MCODE_score in Cytoscape, fold changes, and *P* value in the interaction network in CKD patients compared to healthy controls. (b) mRNA expression of CEBPZ. (c) mRNA expression of IFI16. (d) mRNA expression of LYAR. (e) mRNA expression of BRIX1. (f) mRNA expression of BMS1. (g) mRNA expression of DDX18. CKD: patients with chronic kidney disease; HC: healthy human control.

First, we aggregated the details, including degree, MCODE_score in Cytoscape, fold changes, and *P* value, of these candidate genes and found high scores for these analyses (Figure 5(a)). To validate the key molecules screened by the above bioinformatics, we collected new sequencing data as a validation dataset in GEO. GSE142153 and GSE70528 were used to analyze and validate gene expression. The results showed that these molecules were significantly upregulated in CKD compared to healthy controls (Figures 5(b)–5(g)). Therefore, these molecules, including CEBPZ, IFI16, LYAR, BRIX1, BMS1, and DDX18, can be used as potential candidate markers in CKD.

## 4. Discussion

CKD has a wide range of underlying causes, including both hereditary and environmental factors, and is considered to be a global public health problem, with the adjusted prevalence of CKD in the European adult population ranging from 3.3% to 17.3% [17]. CKD is more common among the elderly and is associated with a higher risk of cardiovascular disease (CVD). One of the most common causes of kidney CKD is diabetes mellitus (DM). In addition, few biomarkers have been found in clinical practice, although other diagnostic biomarkers for CKD have been studied [18]. Therefore, improved early detection and treatment of CKD necessitated new molecular biomarkers.

IFI16 has been shown to influence ribosome biogenesis and has been identified as a promoter associated with stem cell-like properties in colorectal cancer [19]. LYAR can enhance the stem cell-like properties of breast cancer and lead to poor prognosis of breast cancer, which is expected to be a potential biomarker for breast cancer treatment [20]. BRIX1 is a new potential target in psoriasis and diffuse superficial actinic sweat keratosis [21]. BMS1 is an RNA- and DNA-binding protein involved in nucleolar processing of 7S to 5.8SrRNA. When exposed to cytotoxic agents, the nucleolar localization of PUF-A redistributes into the nuclear cytoplasm. DDX18 is a risk site associated with DTC susceptibility [22]. CEBPZ was also involved in cell growth and differentiation, especially hematopoietic differentiation [23]. DDX18 was identified to be associated with stroke, and serum PDCD11-AB levels may serve as a potential biomarker for TRANSIENT ischemic attack [24, 25].

The proliferation of omics-related biomarker studies over the past decade reflects the need for new, effective, noninvasive tools that can identify people at risk for CKD and help target kidney disease management [26]. CKD was a growing public health problem with high morbidity and mortality. New biomarkers were developed to improve risk stratification and clinical decision-making and to guide the enrichment of patients with CKD in clinical trials. Despite tremendous efforts, only a few biomarkers have so far found large-scale clinical application. Although our study has identified multiple putative biomarkers, these were mainly from small, single-center studies. The utility of such biomarkers needed to be confirmed in different populations and in larger cohorts. In addition, no in vivo or in vitro studies were performed. Both the above deficiencies are also the main aspects of our further research.

## 5. Conclusions

We identified 6 hub genes in CKD, which ere demonstrated in the validation dataset. They could be used these molecules as candidate markers to distinguish CKD from healthy people. Our study indicated that 6 hub genes may play key roles in the occurrence and development of CKD.

## Data Availability

The data used to support the findings of this study are available from the corresponding author upon request.
